# Habitat and host factors associated with liver fluke (*Fasciola hepatica*) diagnoses in wild red deer (*Cervus elaphus*) in the Scottish Highlands

**DOI:** 10.1186/s13071-019-3782-3

**Published:** 2019-11-12

**Authors:** Andrew S. French, Ruth N. Zadoks, Philip J. Skuce, Gillian Mitchell, Danielle K. Gordon-Gibbs, Mark A. Taggart

**Affiliations:** 1 Environmental Research Institute, North Highland College, University of the Highlands and Islands, Castle Street, Thurso, KW14 7JD UK; 2 Present Address: Marine Institute, Furnace, Newport, Co. Mayo Ireland; 3 Institute of Biodiversity Animal Health and Comparative Medicine, University of Glasgow, Garscube Campus, Glasgow, G61 1QH UK; 4 Moredun Research Institute, Pentlands Science Park, Bush Loan, Penicuik, EH26 0PZ UK; 5 Present Address: Sydney School of Veterinary Science, University of Sydney, Camden, NSW 2570 Australia

**Keywords:** Wildlife disease, Cervidae, Coproantigen ELISA, Geographical information system, Infection risk factors

## Abstract

**Background:**

Red deer (*Cervus elaphus*) are a common wild definitive host for liver fluke (*Fasciola hepatica*) that have been the subject of limited diagnostic surveillance. This study aimed to explore the extent to which coprological diagnoses for *F. hepatica* in red deer in the Scottish Highlands, Scotland, are associated with variability among hosts and habitats.

**Methods:**

Our analyses were based on coproantigen ELISA diagnoses derived from faecal samples that were collected from carcasses of culled deer on nine hunting estates during two sampling seasons. Sampling locations were used as centroids about which circular home ranges were quantified. Data were stratified by season, and associations between host, hydrological, land cover and meteorological variables and binary diagnoses during 2013–2014 (*n* = 390) were explored by mixed effect logistic regression. The ability of our model to predict diagnoses relative to that which would be expected by chance was quantified, and data collected during 2012–2013 (*n* = 289) were used to assess model transferability.

**Results:**

During 2013–2014, habitat and host characteristics explained 28% of variation in diagnoses, whereby half of the explained variation was attributed to differences among estates. The probability of a positive diagnosis was positively associated with the length of streams in the immediate surroundings of each sampling location, but no non-zero relationships were found for land cover or lifetime average weather variables. Regardless of habitat, the probability of a positive diagnosis remained greatest for males, although males were always sampled earlier in the year than females. A slight decrease in prediction efficacy occurred when our model was used to predict diagnoses for out-of-sample data.

**Conclusions:**

We are cautious to extrapolate our findings geographically, owing to a large proportion of variation attributable to overarching differences among estates. Nevertheless, the temporal transferability of our model is encouraging. While we did not identify any non-zero relationship between meteorological variables and probability of diagnosis, we attribute this (in part) to limitations of interpolated meteorological data. Further study into non-independent diagnoses within estates and differences among estates in terms of deer management, would improve our understanding of *F. hepatica* prevalence in wild deer.

## Background

Liver fluke (*Fasciola hepatica*) risk to grazing ruminants in Europe increased during the 1990s [1] and was reflected in Great Britain (GB) by an increase in the diagnostic rates of liver fluke in sheep and cattle [2, 3]. During the same period, *F. hepatica* occurrence in common wild definitive hosts, such as cervids, received only fleeting attention, e.g. as part of histopathological surveys of red (*Cervus elaphus*) and sika (*Cervus nippon*) deer in Scotland [4].

*Fasciola hepatica* has long-recognised detrimental effects on health and productivity of sheep and cattle [5, 6], and a similar effect might be hypothesised for wild herbivores. More recently, a link between *F. hepatica* infection, decreased immune response and increased risk of infection of cattle by verocytotoxin producing *Escherichia coli* 0157 has been proposed [7, 8]. In light of possible links such as this, coupled with (for example) an outbreak of *E. coli 0157* in autumn 2015 linked to wild sourced venison [9], identification of environments in which wild deer might acquire *F. hepatica* infection is of interest.

The persistence of *F. hepatica* in a definitive host population is dependent on sufficient moisture and warmth (> 10 °C) to facilitate *F. hepatica* egg development, the motility of free-living larval stages and the activity of a locally resident intermediate host, e.g. *Galba truncatula* [10, 11]. Where definitive and intermediate hosts are resident in a temperate climate, e.g. in a large proportion of GB (England and Wales), the summer risk of *F. hepatica* infection is predictable based on the principle that transmission is limited primarily by moisture during the summer months (i.e. the balance between rainfall and evapotranspiration; [12]). However, the extent to which this principle is valid for comparatively drier/wetter or warmer/cooler climates, or for animals where infection of the host is not interrupted by anthelminthic treatment, is unclear. For example, drier regions can experience a negative correlation between *F. hepatica* incidence and rainfall (e.g. in Belgium; [13]), owing to an increase in fresh vegetation growth away from permanent water sources around (and in) which intermediate hosts reside.

Red deer in the Scottish Highlands are exposed to a prevailing wet (extreme in GB context) climate with regional averages of 1700 mm of rainfall and 207 days of rain per year (1981–2010 climate period; [14]). This is in excess of the limitations proposed by Ollerenshaw [15], beyond which the relationship between weather and incidence ceases to be valid. *Fasciola hepatica* prevalence in red deer varies temporally [16] and geographically throughout the Highlands [17]. While geographical variation could be related to *F. hepatica* development in certain areas of the Scottish Highlands being limited by temperature [18], the landscape is markedly different from the pasture dominated rural areas of England and Wales in which temperature-moisture relationships were originally validated. As such, the Scottish Highlands are topographically diverse and offer a range of microclimates and a patchwork of habitats (dominated by heather and blanket bog) that might or might not provide tolerable environments for intermediate hosts, including atypical hosts such as *Radix balthica* [19].

The aim of this study was to use coproantigen ELISA (cELISA) surveillance data from wild red deer faecal samples to explore spatial variation in *F. hepatica* diagnoses in the Scottish Highlands in relation to a suite of host, hydrological, meteorological and land cover variables at the individual level. In so doing, we aim to identify factors associated with *F. hepatica* diagnosis in wild red deer.

## Methods

### Study sites, sampling methods and *F. hepatica* diagnoses

**Fig. 1:**
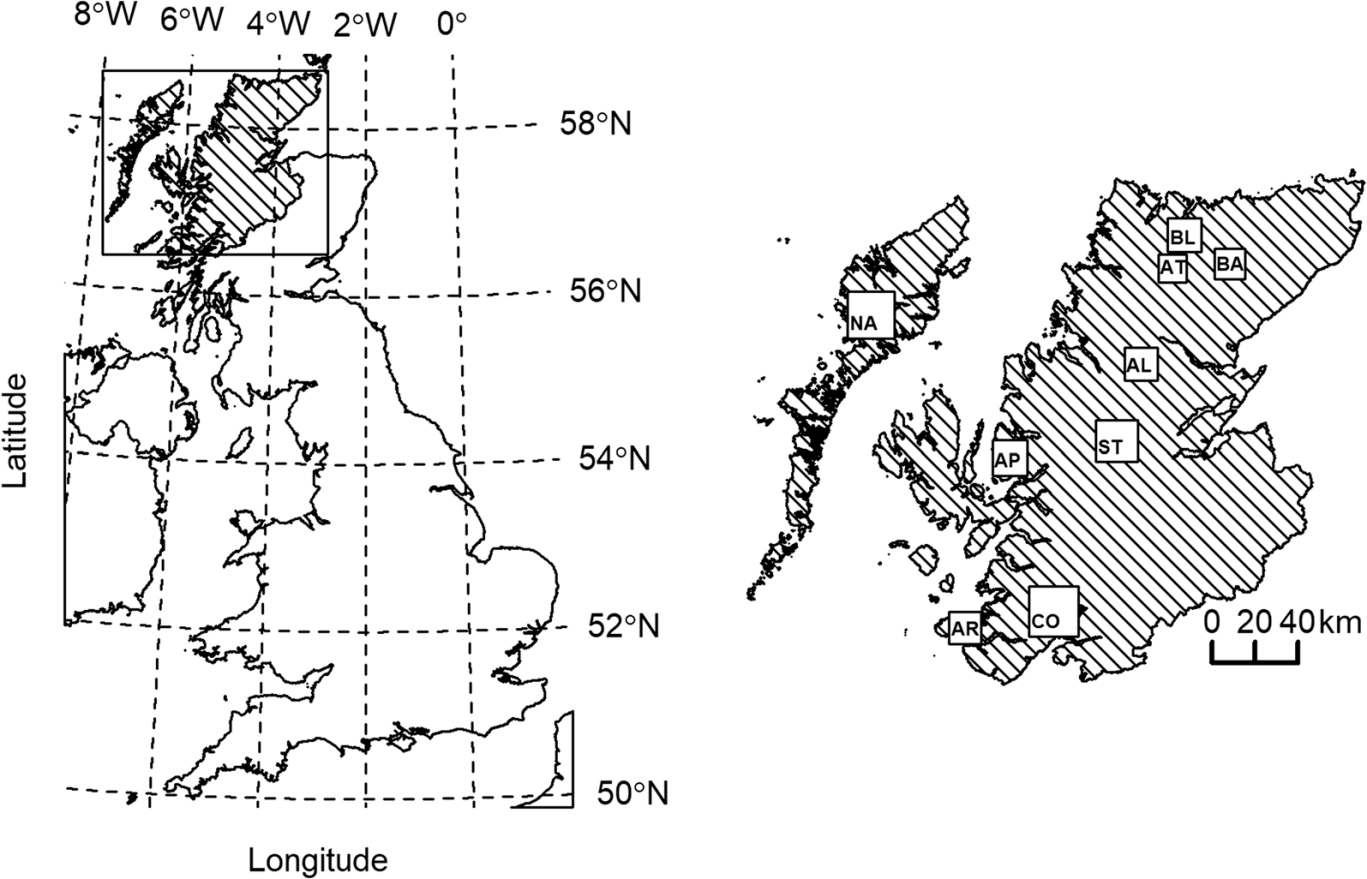
Locations of Scottish Highland hunting estates from which 679 faecal samples were collected from culled wild red deer (*Cervus elaphus*) during August to February 2012–2013 and 2013–2014 for testing by cELISA for *Fasciola hepatica* antigens. *Abbreviations*: AL, Alladale; AT, Altnaharra; AP, Applecross Trust; AR, Ardnamurchan; BA, Badanloch; BL, Ben Loyal; CO, Conaglen; NA, North Harris Trust and Aline; ST, Strathconon. Box sizes illustrate the geographical extent from which samples were collected within each estate. The maps were created using QGIS v2.18.2, R v 3.5.2, RStudio v1.1.383 and the *rgdal* and *PBSmapping* R packages [33, 34] and were produced in accordance with the Public Sector (Scotland) End User License Agreement–1 August 2017–31 July 2018 for the use of Ordnance Survey data through EDINA Digimap (http://digimap.edina.ac.uk/webhelp/os/copyright/licence_agreement.htm#5.1.4)

Red deer faecal samples were collected by deer stalkers/gamekeepers (hunters) on unfenced Scottish Highland estates, which are managed largely for red deer stalking (hunting): Alladale (AL); Altnaharra (AT); Applecross Trust (AP); Ardnamurchan (AR); Badanloch (BA); Ben Loyal (BL); Conaglen (CO); North Harris Trust and Aline (NA); and Strathconon (ST) (Fig. 1; see “Methods” section of [17] for an ethics statement regarding the sampling of wild red deer for this study). During the 2012–2013 and 2013–2014 Scottish red deer stalking (hunting) seasons (for males: 1st July to 20th October; for females: 21st October to 15th February), hunters collected approximately 20 cm^3^ of faecal pellets from the carcasses of 772 red deer and recorded the sex, cull date (which was subsequently converted to an integer number of days after 1st July for each of 2012–2013 and 2013–2014 seasons), age category and sampling location (from a 500 × 500 m grid). Age category was determined (objectively) from tooth eruption for calves and yearlings and (subjectively) from inspection of tooth wear for young, mature and old animals. Across both seasons, 642 faecal samples were stored frozen at − 20 °C on the day of collection until analyses at the end of each season, and a further 130 samples, which were collected during the 2013–2014 season and stored fresh in refrigerators and chilled larders at AT, BA and BL, were analysed no later than one week after the date of collection. A *F. hepatica* coproantigen ELISA (cELISA; BioX Diagnostics, Belgium) (see French et al. [17] for methods) was used to detect antigens specific to *F. hepatica* and each sample was individually classified as positive (exposed to *F. hepatica*) if its titre exceeded the cELISA manufacturer’s cut-off titre, which ranged from 6.55 to 9.32% (ELISA units; EU) of a positive control antigen supplied with the kit. For frozen faeces, the sensitivity of the cELISA to patent infection (using faecal egg identification to define 52 known positives) was 79% (95% CI: 73–84%) and for fresh faeces (using faecal egg identification to define 18 known positives) it was 50% (95% CI: 32–78%). The specificity of the cELISA to patent infection (using liver examination to define 47 known negatives) was 96% (95% CI: 95–100%), whereby all faeces were fresh.

In the interests of balancing our statistical analysis, data for calves and yearlings were removed from the dataset prior to analyses as not all estates had sampled calves and yearlings for both sampling seasons, whereas data for young, mature and old deer were retained and were considered representative of a single class of “independent deer” (*n* = 679; see Table 1 for breakdown of sample sizes related to sex and each sampling season).

**Table 1:** Sample sizes and *Fasciola hepatica* coproantigen ELISA diagnoses for red deer (*Cervus elaphus*) faecal samples collected from nine hunting estates in the Scottish Highlands during two sampling seasons (2012–2013 and 2013–2014)

Estate	Sex	2012–2013				2013–2014			
		*n*	−	+	Prevalence	*n*	−	+	Prevalence
Alladale	F	23	20	3	13.0	29	27	2	6.9
	M	32	25	7	21.9	37	32	5	13.5
Altnaharra	F	29	19	10	34.5	29	25	4	13.8
	M	31	17	14	45.2	28	12	16	57.1
Applecross	F	8	7	1	12.5	16	13	3	18.8
	M	32	17	15	46.9	26	12	14	53.8
Ardnamurchan	F	5	5	0	0	10	8	2	20.0
	M	16	10	6	37.5	25	20	5	20.0
Badanloch	F	21	21	0	0	34	33	1	2.9
	M	25	21	4	16.0	27	23	4	14.8
Ben Loyal	F	22	20	2	9.1	16	14	2	12.5
	M	16	12	4	25.0	24	22	2	8.3
Conaglen	F	7	4	3	42.9	17	11	6	35.3
	M	13	10	3	23.1	17	12	5	29.4
North Harris and Aline	F	0	0	0	NaN	11	2	9	81.8
	M	6	3	3	50.0	2	2	0	0
Strathconon	F	1	1	0	0	17	12	5	29.4
	M	2	1	1	50.0	25	14	11	44.0
All	F	116	97	19	16.4	179	145	34	19.0
	M	173	116	57	32.9	211	149	62	29.4
	Total	289	213	76	26.3	390	294	96	24.6

*Abbreviations*: F, female deer; M, male deer; NaN, not a number

*Key*: +, positive cELISA diagnoses; -, negative cELISA diagnoses

### Geographical information systems and explanatory variable (covariate) preparation

Daily mean temperature (°C) and rainfall (mm) data were obtained for the period 1960–2013 from the UK Meteorological Office’s interpolated 5 km square grid cell dataset [20]. Owing to the disparity between the cross-tabulated format of downloaded meteorological data and the format required for analyses in R [21] (i.e. columns representing variables, rows representing observations), chronological temperature data for 846 grid cells spanning the study region required re-organisation. Data were re-organised by importing downloaded .csv data using the read_csv() function in the *readr* R package [22] and using the melt() function in the *reshape* R package [23]. Next, the subset() function in base R, the cast() function in the *reshape* R package [23] and the between() function in the *data.table* R package [24] were used to obtain 846 grid cell estimates for the (2008–2012 and 2009–2013 meteorological periods) mean number of days per year where temperature exceeded 10 °C (“development days”). Rainfall data were similarly re-organised to obtain 846 grid cell estimates for mean annual rainfall. Topographic photogrammetrically derived (5 × 5 m resolution) digital terrain model (DTM) data were obtained from the UK Centre for Environmental Data Analysis [25] and were used to generate polyline stream networks based on a 4 hectare flow initiation threshold [26] using the Hydrology tools in ArcMap 10.4 [27]. Land cover shapefile data (Land Cover Scotland 1988; LCS88) were downloaded from the UK government website [28] and converted to raster format using Quantum GIS v2.18.2 [29].

**Fig. 2:**
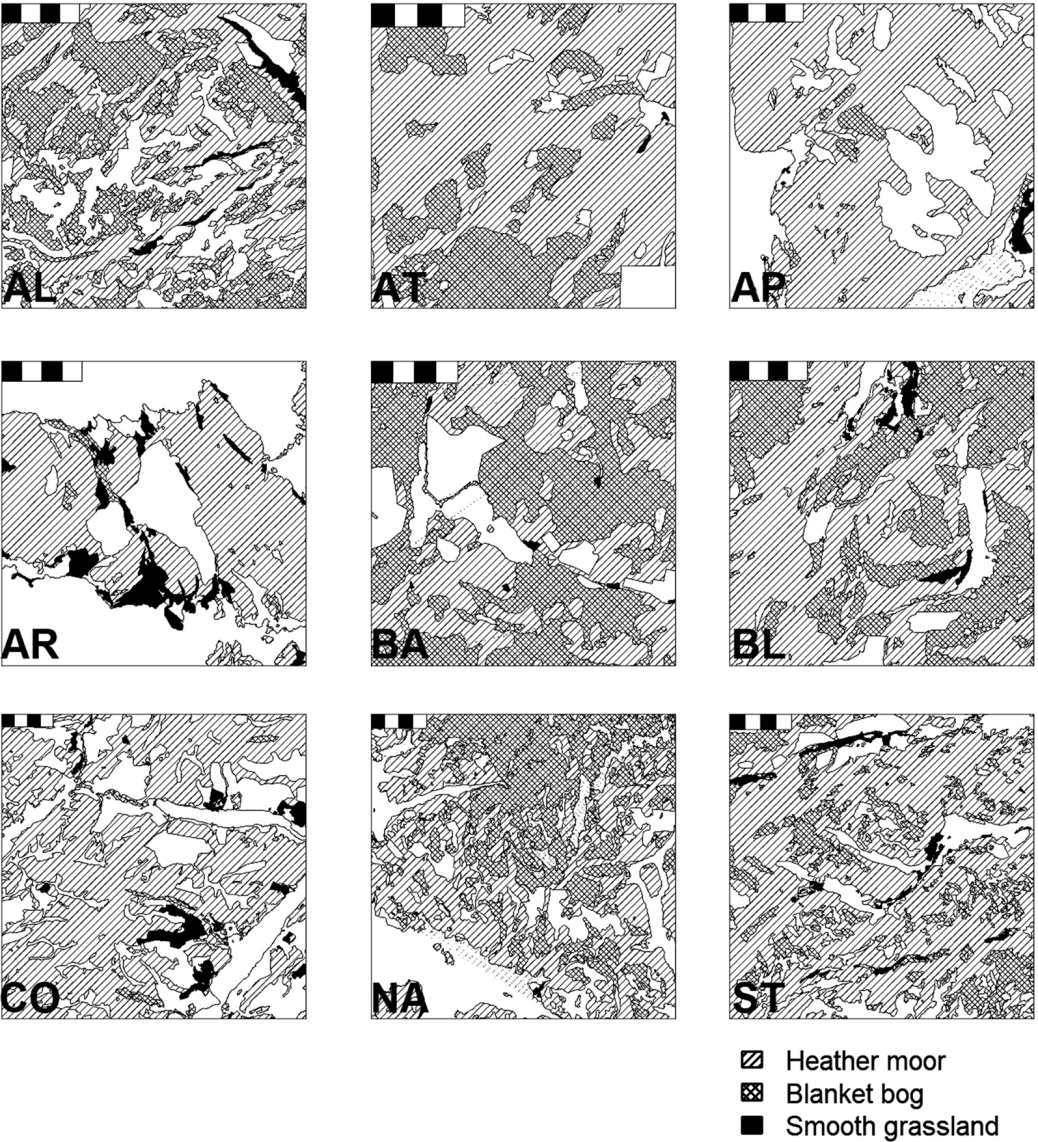
Land cover types on nine hunting estates in the Scottish Highlands. The scale-bar at the top left of each map represents a 4 × 1 km^2^ area. Land cover data (LCS88) were obtained from the UK government [28]. The maps were created using QGIS v2.18.2, R v 3.5.2, RStudio v1.1.383 and the *rgdal* and *PBSmapping* R packages [33, 34] and were produced in accordance with the Public Sector (Scotland) End User License Agreement–1 August 2017–31 July 2018 for the use of Ordnance Survey data through EDINA Digimap (http://digimap.edina.ac.uk/webhelp/os/copyright/licence_agreement.htm#5.1.4). *Abbreviations*: AL, Alladale; AT, Altnaharra; AP, Applecross Trust; AR, Ardnamurchan; BA, Badanloch; BL, Ben Loyal; CO, Conaglen; NA, North Harris Trust and Aline; ST, Strathconon

Covariate data relating to the immediate surroundings of each cull location, i.e. 2 km home range radii (12.6 km^2^) buffers created using the gBuffer() function in the *rgeos* R package [30] were paired with sample diagnoses using R. Home ranges for individuals that had been culled within 2 km of the coast were clipped to the coastline and were delineated as (relatively) smaller home ranges. Hydrological data were extracted using the st_intersection() function in the *sf* R package [31], and land cover and meteorological data were extracted using the extract() function in the *raster* R package [32]. All spatial data were plotted in R using the *rgdal* and *PBSmapping* R packages [33, 34]. Three land cover covariates (smooth grassland, heather moor and blanket bog), which were present in delineated home ranges on all nine estates during both sampling seasons for both sexes, were quantified as the proportion of home range area occupied by each cover type (Fig. 2). One hydrological covariate (stream length) was quantified as the total length of streams within each home range. Two meteorological covariates (rainfall total and development days) were quantified as the mean annual rainfall and mean annual development days experienced across approximate deer lifetimes (i.e. 5 years: 2008–2012 for deer culled during 2012–2013, and 2009–2013 for deer culled during 2013–2014).

### Statistical analyses

All statistical analyses were carried out using R 3.5.2 and RStudio v1.1.383 [21, 35] and we followed Zuur & Ieno’s [36] protocol for conducting and presenting regression type analyses and the worked example of a generalised linear mixed effect model of Elston et al. [37].

Owing to marked environmental gradients in rainfall [38] that inhibited spatially blocked cross-validation [39], we stratified our data by sampling season to obtain a dataset for model training and validation (*n*_(2013–2014)_ = 390; 96 positive cELISA diagnoses, 62 male, 34 female; 294 negative cELISA diagnoses, 149 male, 145 female) and a test dataset to assess the transferability of relationships quantified for the training data (*n*_(2012–2013)_ = 289; 76 positive cELISA diagnoses, 57 male, 19 female; 213 negative cELISA diagnoses, 116 male, 97 female) (i.e. model transferability to an independent dataset; [40]).

Owing to the Bernoulli distribution of our diagnostic response data and the nested structure of our sampling design, we implemented a binomial (logistic) generalised linear mixed effect model (GLMM) using the glmer() function in the *lme4* R package [41]. Here, we assumed that conditional on random intercept, *Estate*_*i*_, and eight covariates, the presence-absence data, *Y*_*ij*_ (*jth* observation on estate *i*), were binomially distributed with a conditional probability *p*_*ij*_, whereby the response *Y*_*ij*_ was 1 if faecal sample *j* on estate *i* was diagnosed positive and was 0 otherwise (Equation 1). The relationship between *p*_*ij*_ and the linear predictor was determined by the logit link function, and the random intercept *Estate*_*i*= *1,* … , *9*_ was assumed to be normally distributed with mean 0 and variance $\sigma_{Estate}^{2}$ (Equation 1).

1 $$ \begin{array}{l} Y_{ij}  \sim  Bin\left( {1,p_{ij} } \right) \\ E\left( {Y_{ij} } \right)  =  p_{ij} \\ logit\left( {p_{ij} } \right)  =  \alpha + \beta_{1} \times sex_{ij} + \beta_{2} \times DateCulled_{ij} + \beta_{3} \times StreamLength_{ij} + \beta_{4} \times BlanketBog_{ij} \\ \quad + \beta_{5} \times HeatherMoor_{ij} + \beta_{6} \times SmoothGrassland_{ij} + \beta_{7} \times RainfallTotal_{ij} + \beta_{8} \times DevelopmentDays_{ij} + Estate_{i} \\ Estate  \sim  N\left( {0,\sigma_{Estate}^{2} } \right) \\ \end{array} $$

Prior to modelling, each continuous fixed effect covariate was mean-centred and scaled to standard deviation units using the scale() function in R, and collinearity between fixed effect covariates were assessed by Pearson’s R and point-biserial correlations using the chart.Correlation() function in the *PerformanceAnalytics* R package [42]. We acknowledged prior to modelling that collinearity would be inherent between sex and sampling date, because the males and females were sampled during separate Scottish hunting seasons. This collinearity was a cause for concern for two reasons. First, we would expect inflated variances for sex and sampling date model coefficient estimates. Secondly, we could not be sure whether any observed non-zero associations that each of these variables might have with *F. hepatica* diagnostic probabilities (for our dataset) would reflect true underlying associations in nature had samples for both sexes been collected concurrently. Nevertheless, sex (as a binary variable defined as: males = 1 and females = 0) and sampling date were included in our model owing to their assumed meaningful associations with infection, both in terms of red deer feeding behaviour (i.e. chance of encountering *F. hepatica* metacercariae) and *F. hepatica*’s life-cycle (i.e. seasonal infection/development and detectability of excretory-secretory antigens within definitive host faeces).

To validate our model, we used the *DHARMa* R package [43] to simulate scaled (standardised) model residuals using the simulateResiduals() function, which we then visually inspected versus fitted values, *versus* each fixed effect covariate, versus time and in space. Our model was deemed valid if standardised residuals were uniformly distributed showing no trends *versus* fitted values, and if plotting residuals against time and in two-dimensional space revealed no clear trends or clusters, respectively.

We assessed model prediction efficacy using block cross-validation [39]. Here, we used the roc() and auc() functions in the *pROC* R package [44] to calculate the area under the receiver operating characteristic curve (AUC). Using the AUC, we inferred the model’s ability to correctly assign high probabilities to positive cELISA diagnoses and low probabilities to negative diagnoses for the training and testing datasets. Here, we used the definitions provided by Swets [45] to define models of low accuracy (AUC 0.50–0.70) and useful accuracy (AUC 0.70–0.90). We also applied Swets’ [45] interpretation that the AUC corresponds to the percentage of the time that, for a given randomly selected positive and a randomly selected negative diagnosis, our model predicts a higher probability for the positively diagnosed sample (bearing in mind that an AUC of 0.50 would be calculated for a model that is no better than would be expected by chance). We also estimated the sensitivity and specificity of the model, whereby we used a predicted probability threshold of 0.50 to signify positive predictions.

Consistent with the logistic and random intercept form of our model, we estimated effective sample size, N_eff_, (Equation 2), and with it, our model’s inclination towards over parameterization and therefore overfitting [46]. This calculation required: (i) an estimate of random intercept variance, $\sigma_{Estate}^{2}$, to calculate the (induced) intra-class correlation coefficient, ICC [47] (i.e. a quantification of non-independence of events within sampling estates, or “compound symmetry”; Equation 3); (ii) the number of random effect levels in our training data, *N*, (here, *N* = 9 estates); and (iii) the number of events per estate, *n* (here, *n* = 10.7; i.e. assuming 96 positive diagnoses are spread evenly across all nine estates). In advance of model fitting, we calculated an ICC of no greater than 0.021 would correspond with a large enough effective sample size to satisfy the recommended minimum of ten events per variable that would mitigate risk of overfitting a logistic regression type model [48]. Following model training, we used the icc() function in the *sjstats* R package [49] to corroborate our ICC (as calculated using Equation 3). While our modelling approach ensured that parameter estimates provided by glmer() in *lme4* defaulted to Laplace approximation and thus provided a point estimate of $\sigma_{Estate}^{2}$, we obtained a 95% confidence interval for $\sigma_{Estate}^{2}$ by using the confint.merMod() method in the *lme4* R package [41], whereby we used profile intervals to propagate uncertainty for the ICC. Note that increasing quadrature points using glmer(nAGQ = 50) did not change model coefficient estimates.

2 $$ N_{eff} = \frac{{\left( {N \times n} \right)}}{{\left( {1 + \left( {n - 1} \right)ICC} \right)}} $$

3 $$ ICC = \frac{{\sigma_{Estate}^{2} }}{{\left( {\sigma_{Estate}^{2} + \left( {\frac{{\pi^{2} }}{3}} \right)} \right)}} $$

To help us to evaluate model transferability (i.e. to give context to any differences between the prediction efficacy of the model that occurred between training (2013–2014) and test (2012–2013) data), we estimated the influence of data on model parameter estimates attributable to each individual observation and to observations grouped by estate. Here, we used the influence() and cooks.distance() functions in the *influence.ME* R package [50] to calculate the Cook’s distance summary statistic. The strongest influence attributable to data related to individual observations or random effect levels was identified by the largest Cook’s distances.

The variability in the data captured by our model was estimated using marginal and conditional R^2^ [51] using the rsquared() function in the *piecewiseSEM* R package [52]. With the exception of the categorical variable, sex (for which an odds ratio was calculated by exponentiating its model coefficient estimate), strengths and directions of relationships between fixed effect covariates and probability of *F. hepatica* diagnosis (i.e. effect sizes) were inferred from the magnitudes and directions of model parameter estimates. Moreover, non-zero relationships between diagnostic probabilities and each fixed effect covariate $\left( {x_{k = 1, \ldots ,8} } \right)$ (estimated using the “profile” confint.merMod() method in the *lme4* R package [41]) and their respective 95% confidence intervals for each sex were illustrated using logistic regression (Equation 4) and an adapted version of the logi.hist.plot function in the *popbio* R package [53].

4 $$ \begin{array}{*{20}c} {p = \frac{{e^{{\alpha + \beta_{k} x_{k} \pm 1.96\left( {\sigma_{Estate} } \right)}} }}{{1 + e^{{\alpha + \beta_{k} x_{k} \pm 1.96\left( {\sigma_{Estate} } \right)}} }}} \\ \end{array} $$

## Results

### Descriptive analysis of spatial aggregation in *F. hepatica* diagnosis

**Fig. 3:**
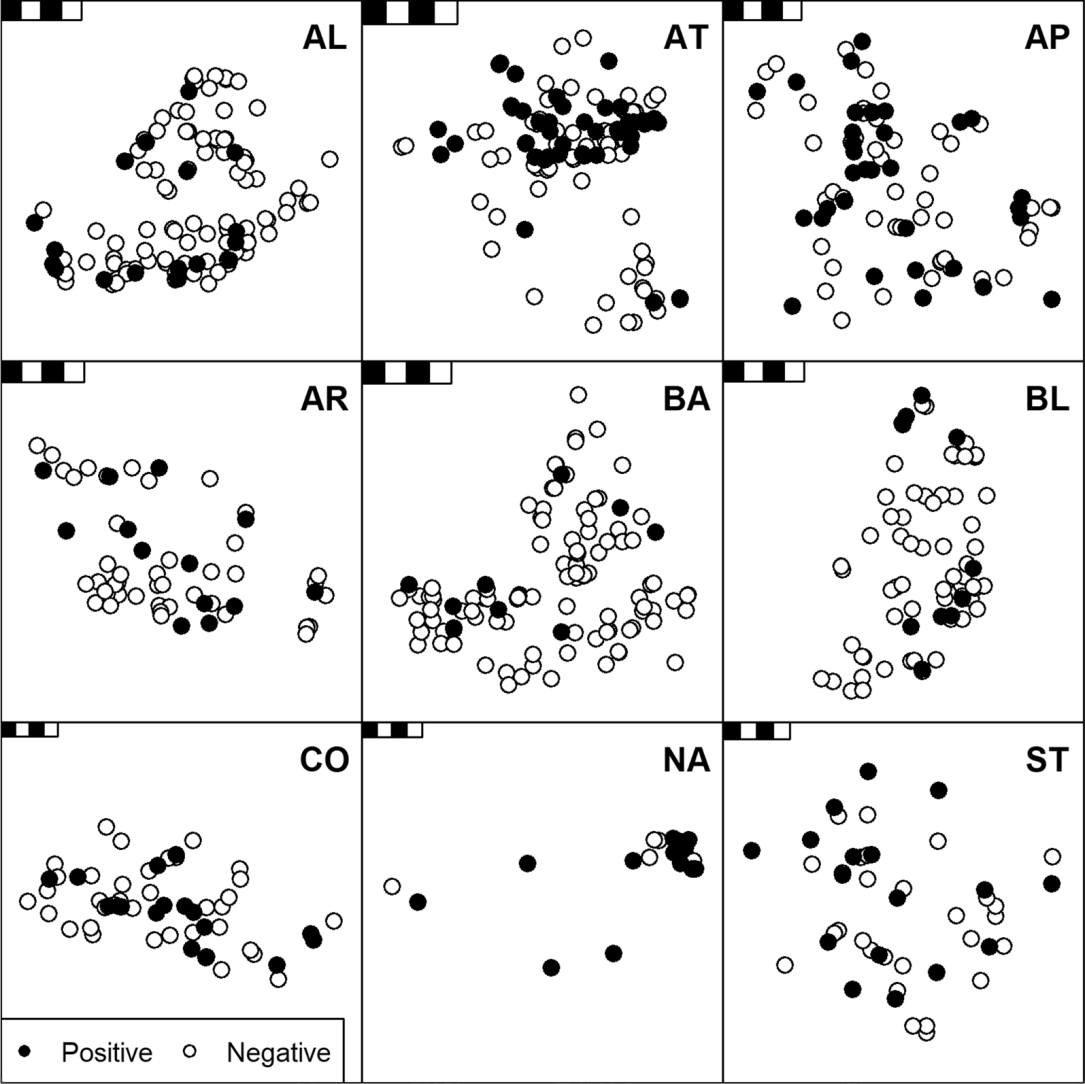
Spatial distribution of *Fasciola hepatica* coproantigen ELISA diagnoses for 679 wild red deer (*Cervus elaphus*) faeces samples collected within nine Scottish Highland hunting estates during 2012–2014. Filled circles represent positive diagnoses. Random noise has been added to the x-y coordinates for each data point to aid illustration of diagnoses where more than one sample was collected at a single location. Scale-bars illustrate 4 × 1 km areas for each estate. *Abbreviations*: AL, Alladale; AT, Altnaharra; AP, Applecross Trust; AR, Ardnamurchan; BA, Badanloch; BL, Ben Loyal; CO, Conaglen; NA, North Harris Trust and Aline; ST, Strathconon

Positive *F. hepatica* cELISA diagnoses generally displayed a random spatial distribution relative to negative diagnoses (Fig. 3).

### *Fasciola hepatica* diagnosis in relation to habitat and host variation

#### Data exploration and model validation

A full list of explanatory variables and their ranges for males and females for each sampling season is presented in Table 2. Only one correlation between two fixed effect covariates exceeded the |R| > 0.70 stipulated by Dormann et al. [54] as a warning as to when multicollinearity might affect the stability of regression parameter estimates; unsurprisingly, this was the point-biserial correlation (|R| = 0.81; 95% CI: 0.77–0.84) between sex and cull date.

**Table 2:** Summary of host and habitat characteristics for wild red deer (*Cervus elaphus*) from estimated 2 km radius home ranges in the Scottish Highlands. Units in which ranges are expressed are indicated in parentheses in the explanatory variables column

Explanatory variable	2012–2013		2013–2014	
	M (*n* = 173)	F (*n* = 116)	M (*n* = 211)	F (*n* = 179)
Days into season (days)	33–112	114–230	39–112	113–228
Stream length (km)	24.7–62.6	22.2–64.9	24.1–64.5	20.8–65.2
Blanket bog (%)	0–86.8	0–72.4	0–90.5	0–81.4
Heather moor (%)	13.2–99.9	18.6–94.5	8.2–97.6	10–99.7
Smooth grassland (%)	0–42.5	0–40.2	0–39.3	0–50.6
Mean annual rainfall total (mm)	1053–3363	1005–3715	1002–3564	1013–3658
Mean annual development days (days)	35.5–163.9	35.5–157.3	40–157.7	37–156.9

*Abbreviations*: F, female deer; M, male deer

*Notes*: Annual development days were defined as the total number of days per year with mean air temperature exceeding 10°C. Mean annual rainfall total and mean annual development days were calculated using data from the 5 years immediately preceding each sampling season

Model validation revealed no strong non-linearity (Additional file 1: Figure S1), residual temporal autocorrelation (Additional file 2: Figure S2), nor residual spatial autocorrelation (Moran’s I: Expected = −0.0026, Observed = 0.041, *P* = 0.22) (Additional file 3: Figure S3). Our assumption of linear relationships between model covariates and the logit link transformed expectation of the response were validated by residual plots, though we noted a possible borderline non-linear relationship for smooth grassland (Additional file 1: Figure S1).

#### Model parameter estimates, prediction efficacy, variance and transferability

**Fig. 4:**
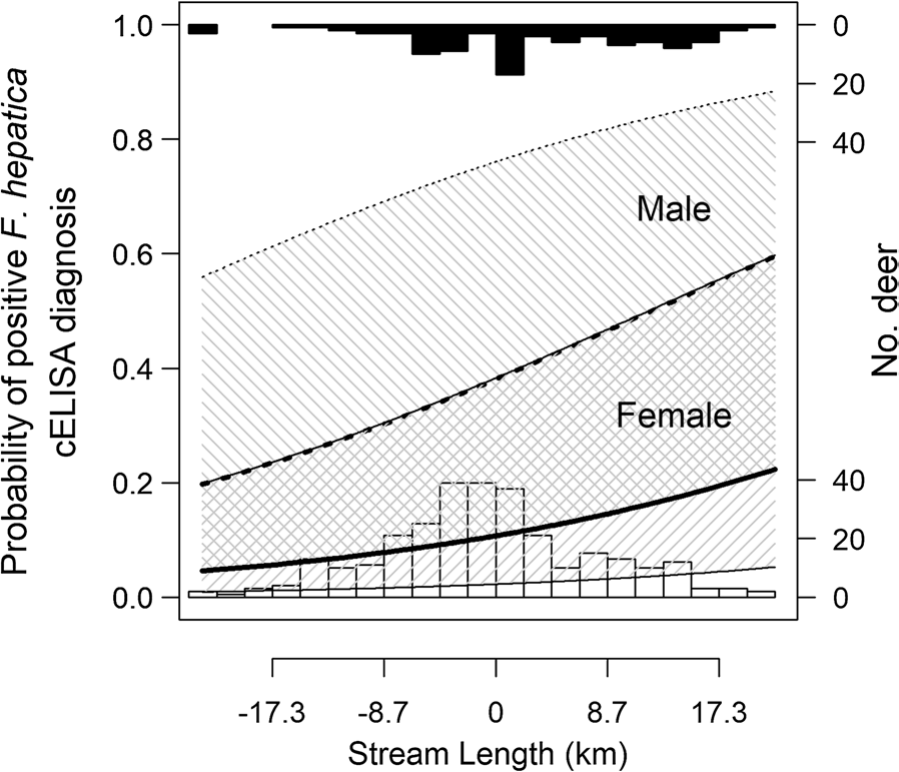
Visual representation of a generalised linear mixed effect model parameter estimate describing probability of positive *Fasciola hepatica* coproantigen ELISA diagnosis in wild red deer (*Cervus elaphus*) in relation to stream length. Model covariates were mean-centred and scaled to standard deviation units prior to modelling, but axes labels are back-transformed to aid interpretation. This plot illustrates the probability of a positive diagnosis for a sample collected from a deer with a home range stream length exceeding (x > 0), or less than (x < 0), a deer that occupies “average habitat” (x = 0). Relationships for males (dashed) and females (solid) are illustrated with thick lines; thin lines indicate 95% (Wald) confidence intervals calculated using the random effect standard deviation. Histograms at the top (upside down filled) and bottom (unfilled) of the plot illustrate the number of deer for which faecal samples were allocated a positive and negative cELISA diagnosis, respectively

The total variation in diagnostic probabilities explained by the combination of random and fixed effects was 28% (conditional *R*^2^). Only 13% (marginal *R*^2^) of the variation was explained by fixed effects alone and only one habitat related fixed effect parameter estimate was non-zero (Table 3). Here, a strong positive association occurred for stream length (Fig. 4). We found no non-zero associations between diagnosis and land cover or meteorological variables.

**Table 3:** Fixed effect parameter estimates for a binomial (logit link) generalised linear mixed effect model describing the probability of positive *Fasciola hepatica* coproantigen ELISA diagnosis for faecal samples of wild Scottish red deer (*Cervus elaphus*) (*n* = 390) in relation to host and habitat within 2 km radius estimated home range

Fixed effect parameter	Estimate (95% CI)
(Intercept)	− 2.12 (− 3.076 to − 1.198)
Sex	1.634 (0.603–2.722)
Days into season	0.521 (0.016–1.037)
Stream length	0.349 (0.047–0.658)
Mean annual rainfall total	0.148 (− 0.421 to 0.683)
Mean annual development days	0.266 (− 0.179 to 0.708)
Blanket bog	0.115 (− 0.437 to 0.665)
Heather moor	0.223 (− 0.193 to 0.653)
Smooth grassland	0.223 (− 0.082 to 0.527)

*Notes*: Habitat data in terms of home range percentage land cover [28], meteorology [20] and topographically derived stream length [25, 26] were mean-centred and scaled to standard deviation units prior to logistic regression against binary cELISA diagnoses. Values in parentheses represent 95% (profile) confidence intervals. Annual development days were defined as the total number of days per year with mean air temperature exceeding 10 °C. Mean annual rainfall total and mean annual development days were calculated using data from the 5 years immediately preceding sampling season 2013–2014

Of the collinear host-related variables (sex and date culled), the model portioned the strongest non-zero effect size to sex. Here, males were more likely to be diagnosed positive, which equated to an odds ratio relative to females of 5.12 (95% CI: 1.83–15.22) (Table 3). The model also assigned a further (but weaker) non-zero parameter estimate to a positive association with the number of days into the hunting season.

In terms of model prediction efficacy, AUC for the training data indicated useful accuracy (0.78; 95% bootstrap CI: 0.73–0.83). Using the training data and a cut-off probability of > 0.50 as an indicator of positive diagnosis (as predicted by the model), we calculated model sensitivity of 0.23 (95% bootstrap CI: 0.15–0.32) and specificity of 0.96 (95% bootstrap CI: 0.94–0.98).

In terms of model transferability, we considered effective sample size and influential points/groups in the training data in relation to model prediction efficacy using the test data. First, the random effect variance, $\sigma_{Estate}^{2}$, for the model 0.70 (95% CI: 0.36–1.70) corresponded to an (induced) intraclass correlation coefficient (ICC) of 0.18 (95% CI: 0.099–0.34), which exceeded our threshold of 0.021 for mitigating against model over parameterization and served as a warning of a possibly insufficient effective sample size for our number of fixed effect covariates. Secondly, we found the strongest influence (inferred from Cook’s distance, D) on model parameter estimates at the group level for NA estate (Cook’s D = 0.58) and the individual level for a positively diagnosed sample collected from a female at AR (Cook’s D = 0.085). Our assessment of model transferability using the test data revealed a reduction in prediction efficacy inferred to AUC (0.71; 95% bootstrap CI: 0.64–0.77) compared to our assessment using the training data. Estimates for model sensitivity 0.24 (95% bootstrap CI: 0.14–0.34) and specificity 0.92 (95% bootstrap CI: 0.88–0.95) using the test data were marginally lower and higher, respectively, than estimates using the training data.

## Discussion

This study explored the associations between habitat and host related variables and *F. hepatica* diagnosis in wild red deer in the Scottish Highlands during two sampling years, 2012–2013 and 2013–2014. In terms of habitat variation, we revealed that cELISA based estimates of exposure are most strongly (and positively) associated with home range stream length. Contrary to our expectations, diagnosis did not vary with the annual number of days during which the temperature threshold for *F. hepatica* development was exceeded, nor did diagnosis associate with variation in annual rainfall. Inherent collinearity between sex and sampling date inflated the variance of our model coefficient estimates for these variables, yet we found a strong association between diagnosis and sex (i.e. samples from males had greater odds of being diagnosed positive than did females, regardless of habitat variation), and a weak positive association between diagnosis and sampling date. Both of these relationships should however be interpreted with caution, owing to temporally separated hunting seasons for males and females. Our model explained just over one quarter of the variation in the probability of *F. hepatica* diagnosis; wherein more than half of the explained variation was attributable to unquantified overarching variation among estates (e.g. factors such as hunting biases), and the other just less than half was attributable to individual-level host and home range habitat variation. Our model was useful for predicting diagnoses for 2013–2014, though we observed a slight reduction in model prediction efficacy when using held-out 2012–2013 data, which highlighted the moderate but nonetheless useful transferability of our findings.

### *Fasciola hepatica* diagnoses in relation to habitat and host variation

The relationship between stream length and *F. hepatica* diagnosis might reflect a greater availability of tolerable habitat for an intermediate host; even though the streams themselves are not necessarily occupied by those intermediate hosts. Surveys of macro invertebrates in Scottish Highland running freshwaters reveal that *G. truncatula* is typically absent from streams; i.e. it was found in just one river in our study region between 2005 and 2007 [55, 56]. Notwithstanding possible systematic reasons for this lack of detection (i.e. related to kick sampling methods), it is more plausible that *G. truncatula* simply occurs in habitats close to streams rather than in the streams themselves [57]. Indeed, a range of permanent and temporary small water bodies are tolerable for *G. trunctula* [58].

The lack of association between mean annual rainfall and/or number of development days, and, probability of *F. hepatica* diagnosis, might be a consequence of the large uncertainties that accompany interpolations of meteorological data across topographically diverse regions. In a UK context, grid data is spatially interpolated from station data using a linear relationship between altitude and temperature [59, 60]. However, the 5 × 5 km grid used is likely to be of insufficient scale to resolve differences in weather experienced by deer within a single estate which may only span a maximum of around 15 km (at its greatest extent). Of course, meteorological suitability for *F. hepatica* and its intermediate snail hosts does not necessarily directly imply infection of definitive hosts. *Fasciola hepatica* must first be present, either endemically or introduced (perhaps through livestock imports, as has occurred in historically *F. hepatica*-free areas in the south east of GB; [61]). Additionally, one must consider the tolerability of land cover for intermediate hosts. In this regard, *G. truncatula* is apparently unable to tolerate blanket bog environments (e.g. in Orkney; [10]), which cover large areas of the Scottish Highlands. We note that average annual rainfall total and mean temperature are associated with *F. hepatica* related condemnation of cattle liver at slaughter in Scotland [62], but that the distribution of sampled farms in the Scottish Highlands is concentrated at the coastlines; hence, the rainfall experience by cattle is unlikely to be as great as experienced by the deer in our study, which occupy higher ground primarily inland from the coast.

The lack of relationship between *F. hepatica* diagnosis and the presence of smooth grassland (or indeed any other land cover) within the deer home ranges here, might be an indication that *G. truncatula* has a wide tolerance for other habitats (e.g. in terms of soil pH; as also noted in Orkney; [10]). Alternatively, *G. truncatula* might not be the only important intermediate host, and intermediate hosts that are more tolerant of upland environments may occupy a more prominent role in the *F. hepatica* life-cycle in the Scottish Highlands than is typical in lowland habitats. This latter hypothesis is supported by 19 records of a less common host, *R. balthica* [19, 63, 64], identified during kick sampling of streams in peatland and heather dominated habitats [55, 56].

While we could not disentangle associations between sex and sampling date, their relatively strong explanatory contribution (here mostly weighted to a large effect size for sex; when compared to those estimated for land cover and meteorological variables) alluded to at least one or the other’s substantial association with *F. hepatica* diagnoses. Where these effects have been disentangled using year-round coprological surveys of male and female wild Scottish red deer [16], male biased *F. hepatica* diagnosis has not been observed, whereas seasonal variation in prevalence and intensity of *F. hepatica* diagnoses appears strong. In this case, significantly lower prevalence and intensity of infection (faecal egg counts) occurred in adults of both sexes during winter (i.e. our female sampling season) [16]. The fact that any temporal signature is detectable despite the lack of anthelmintic treatment of wild deer begs further questions. For example, to what extent are diagnoses during a given hunting season a reflection of infection in the previous year, as opposed to lifetime burden? Or, do our observations reflect temporal changes in *F. hepatica* coproantigen detectability, perhaps related to physiological changes such as fatty liver, which occurs in males following the breeding season [65]? If, conversely, our coefficient estimates reflect a true underlying male biased parasitism, this would reflect observations in other wild ungulates (e.g. lungworm (Protostrongylidae) in red deer [66] and chamois (*Rupicapra rupicapra rupicapra*) [67]).

### Variance explained and transferability of findings

We were aware that the explanatory power of regression type analyses inevitably increases with the addition of variables, so we had to ensure we did not overparameterize and thus overfit our model. Various methods to assess the transferability of model findings related to *F. hepatica* exposure in livestock and environmental variability have been explored. For example, using linear regression and a range of explanatory environmental variables, Howell et al. [8] used comparisons of *R*^2^ between training and test data to assess transferability, whereby their fitted model explained 37% of differences in *F. hepatica* exposure among farms in England and Wales, which notably increased to 49% using held-out test data. In Northern Ireland, transferability of findings has been assessed by constructing multiple models and comparing their coefficient estimates, i.e. identifying the most parsimonious model for each year within their study (based on lowest value of the Akaike’s information criterion). There, Byrne et al. [68] noted that separate models for 2011, 2012 and 2013 data that described variance in a binary response variable (*F. hepatica* severity based on cattle herd level prevalence) inconsistently retained long-term weather variables. In the present study, the transferability of our model was encouraging owing to only a slight reduction in point estimates of prediction efficacy for our model when applied to held-out data from 2012–2013. Nevertheless, this demonstrates that our estimate of explained variance (*R*^2^ of 28%) might be marginally over-optimistic.

The amount of variance attributable to unmeasured differences among estates warrants further attention; in this regard, a range of finer scale habitat covariates might improve the explanatory power of these models. However, it is perhaps management related factors (such as supplementary feeding, which is practiced widely in the Scottish Highlands [69]), that could influence (amongst other factors) deer habitat use and spatial aggregation [70] and might thus facilitate the greatest gains in model performance. Unfenced livestock, which are also present in some Scottish Highland hunting estates, are also feasible contributors to *F. hepatica* infection risk for wild deer (and *vice versa*).

### Estate scale non-independence in diagnoses

Any spatial (e.g. Fig. 3) and temporal dependencies in the raw data were accounted for by our model specification. However, in terms of spatial dependency, our (induced) intraclass compound symmetry correlation structure demonstrated that diagnoses within sites were not necessarily independent from each other. While a compound symmetry structure implies that spatial dependencies act equally as strongly over short distances as they do over tens of kilometres (e.g. between two animals on opposite extremities of the same hunting estate), this is a simplified picture. Because of a lack of significant Moran’s I or spatial patterns in residuals, we did not attempt to explicitly quantify distances over which underlying epidemiological neighbourhood scale processes act (i.e. between animals with overlapping home ranges). However, should future surveillance data be of sufficiently high resolution, it might be possible to gain an insight into such processes, as advocated in terms of spatial [13] and temporal dependencies in *F. hepatica* infection and indeed for parasitology in general [71].

### Limitations

Ideally, our surveillance data would have mirrored a fully-crossed (full-factorial) randomized block experimental design insofar as all values for host and environmental explanatory variables would have been replicated on all sampled hunting estates [72]. However, differences in management practices meant that some estates (for example) culled females until late December, while others continued culling until mid-February. Furthermore, we did not include finer scale habitat information, such as *Juncus* spp. infested grassland, which is present in LCS88 and is a useful indicator of *G. truncatula* presence [73], because it was not present on all estates. While our random intercept approach went some way to dealing with our partially-crossed design, it left considerable room for influential estates, i.e. estates for which exclusive combinations of explanatory and response variable values were present. For example, NA estate had the greatest influence on the date culled parameter because more than half of the diagnoses were positive and samples at NA were collected an average of 20 days later than the next latest sampling estate, CO. Similarly, a positively diagnosed sample at AR had the greatest influence on the smooth grassland parameter (albeit ultimately a zero-effect parameter) owing to the large proportion of smooth grassland in its home range.

The effective sample size of our model training data must be considered in relation to two aspects of statistical power. First, for an exploratory study such as ours, power to detect a hypothesised effect size for an explanatory variable is calculated from the combined influence of the magnitude of effect size, the number of samples per hunting estate, the number of estates, and the properties of the variable of interest (e.g. bounded land cover proportion; continuous stream length). Here, our study detected a positive association between *F. hepatica* diagnosis and the length of stream within red deer home range. Were this effect size to be arbitrarily classified as “medium” (for example, owing to a coefficient estimate of ~0.4 standard deviation units), it is important to note that the size of this effect might be overestimated (positively biased) if the power of the design to identify such effects is low (e.g. as demonstrated by simulation studies [74]). Regardless, the confidence intervals (Table 3 and Fig. 4), serve as a warning as to the precision of this effect size estimate.

The second aspect of statistical power to consider is the possibility of Type II error, i.e. does percentage of smooth grassland occupying home ranges of red deer really not associate with the probability of *F. hepatica* diagnosis, or did our design have insufficient power to detect an effect? Relative to stream length, it appears likely that any underlying association between *F. hepatica* diagnosis and smooth grassland is small. To provide an indication of possible Type II error, we retrospectively estimated the power of our analysis (using the *simr* R package [75]) to detect a defined small effect size (0.2 SD) for grassland. The resulting estimate of 32% power based on 250 simulations is undesirable, but nevertheless must be considered in the context of the aim of the study. Our study was exploratory and dependent on sample collections from wild deer, rather than based on controlled field manipulation whereby a design could test for particular sizes of effects (e.g. small, ≤ 0.2 SD), though tools and guidance for *a priori* simulation based power analysis for GLMMs have become available since study [75, 76]); thus, while our results should be considered inconclusive with regards to a possible small association between smooth grassland percentage and *F. hepatica* diagnosis, we can nevertheless have confidence that had there been a larger effect (e.g. 0.5 SD, again tested using the *simr* R package [75]), we had 93% power to detect it.

Our delineated home ranges, while roughly reflecting those for male red deer inhabiting the south west Scottish Highlands (4–30 km^2^) [77], were nevertheless estimates and home ranges may not be consistent across all our study sites. For example, home range estimates for Scottish island (or coastal) dwelling female red deer may be relatively smaller (at mean ± SD; 1.3 ± 1.6 km^2^) than our estimates [78]. In addition, our data collection spanned only seven months of each sampling year and it is unclear whether cull locations accurately represent the centroid of each individual’s annual home range. This is because red deer have different winter and summer ranges depending on (for example) sex and broad-scale habitat type [79]. While more precise knowledge of habitat use by individual deer might have improved our model performance (i.e. prediction efficacy and transferability), it is nevertheless worth noting that Scottish red deer are less selective in terms of grazing patch sizes than other herbivores such as sheep, which tend to prefer smaller patches [80]; thus, proportions of immediate surrounding land cover (as quantified herein) might indeed usefully describe variation in wild red deer habitat related to (for example) a greater probability of encountering infective metacercariae.

The consequences of over- or underestimating red deer home ranges in this study might introduce bias to parameter estimates. For example, if female ranges were overestimated, we would in turn be overestimating the length of stream in their habitat. As such, fewer samples from females than males were diagnosed positive; therefore, it is possible that a portion of the negative diagnoses clustered with relatively long home range stream lengths (e.g. in Fig. 4) were derived from female samples (see similar ranges for both sexes in Table 2) and may therefore be deflating the effect size. This is just one example, but it is important to be aware of the consequences of inaccurate home range estimates in exploratory studies of this type.

Finally, we note that owing to the sensitivity of the cELISA, a portion of positive infections will have been missed and this is likely to have diluted the strength of effects identified by our model.

## Conclusions

The probability of *F. hepatica* diagnosis in Scottish Highland deer is ostensibly associated with variation between the sexes and/or the date on which samples are collected, and the length of streams in an individual’s home range. The association between stream length and diagnosis might be related to the suitability of habitat for intermediate snail hosts (with stream length perhaps acting as a useful proxy related to the likelihood of exposure and thus infection). However, the prevailing importance of various possible intermediate host species requires further investigation. Greater odds of positive diagnosis in males relative to females reflects observations in other ungulates; and, whilst sex was inherently correlated with sampling date (thus inflating the variances associated with our model coefficient estimates for these two variables), this served to demonstrate that the association between at least one of these variables and diagnosis of *F. hepatica* is strong. While we did not identify any non-zero relationship between meteorological variables and probability of diagnosis, we attribute this (in part) to uncertainty in the modelling of climate data in topographically diverse regions. The statistical power of the model was probably limited by a deflated effective sample size and as such we advocate a wider geographical sampling protocol (no more samples per site, but more sites). More than half of the explained variation in diagnosis probability was attributable to unquantified overarching differences among sampling sites, which highlights significant gaps in basic data (i.e. regarding habitat and deer management), and in our understanding. Extensions to explore neighbourhood-scale processes affecting *F. hepatica* transmission could be applied to this study, such as intensive sampling in areas with low *F. hepatica* prevalence. We would then advocate a similar approach to that taken here, in which confidence in the transferability of findings should be mediated by an evaluation of any explanatory model in terms of prediction efficacy and transferability.

## Supplementary information

**Additional file 1: Figure S1.** Standardized residuals *vs* predictors. This figure revealed no strong evidence of non-linear relationships between predictors and response (probability of diagnosis). The plot was produced using the plotResiduals() function in the *DHARMa* R package [43].

**Additional file 2:** Residuals *vs* time (mean-centred and standard deviation scaled days after 1st July 2012) (left), autocorrelation function (ACF) for diagnostic data *vs* time lags of up to 25 days for model residuals (right). The dashed lines in the ACF plot illustrate the magnitude of the autocorrelation function beyond which autocorrelation is statistically significant. Therefore, this figure reveals borderline residual temporal autocorrelation at 1- and 8-days lag; neither of which we consider to be of concern. The figure was produced using the testTemporalAutocorrelation() function in the *DHARMa* R package [43] and the acf() function in the *ncf* R package [81].

**Additional file 3: Figure S3.** Spatially plotted residuals. This figure revealed no evidence of residual spatial autocorrelation and was created using an adaptation of the testSpatialAutocorrelation() function in the *DHARMa* R package [43]. The colour scale illustrates the magnitude of scaled simulated uniform residuals.

## Data Availability

Data supporting the conclusions of this article are provided within the article and its additional files. The datasets analysed during the present study are available from the corresponding author upon reasonable request.

## Abbreviations

ACF: autocorrelation function
AL: Alladale
AP: Applecross Trust
AR: Ardnamurchan
AT: Altnaharra
AUC: area under the receiver operating characteristic curve
BA: Badanloch
BL: Ben Loyal
cELISA: coproantigen enzyme-linked immunosorbent assay
CO: Conaglen
DTM: Digital Terrain Model
GB: Great Britain
GLMM: Generalised Linear Mixed Effect Model
ICC: intraclass correlation coefficient
LCS88: Land Cover Scotland 1988
NA: North Harris Trust and Aline
(Q)GIS: (Quantum) Geographic Information System
ST: Strathconon
