# Supplementary figures and images for: Habitat and host factors associated with liver fluke (Fasciola hepatica) diagnoses in wild red deer (Cervus elaphus) in the Scottish Highlands
Source: Parasit Vectors. 2019 Nov 12;12:535. doi: 10.1186/s13071-019-3782-3 (PMC6852960; doi:10.1186/s13071-019-3782-3)

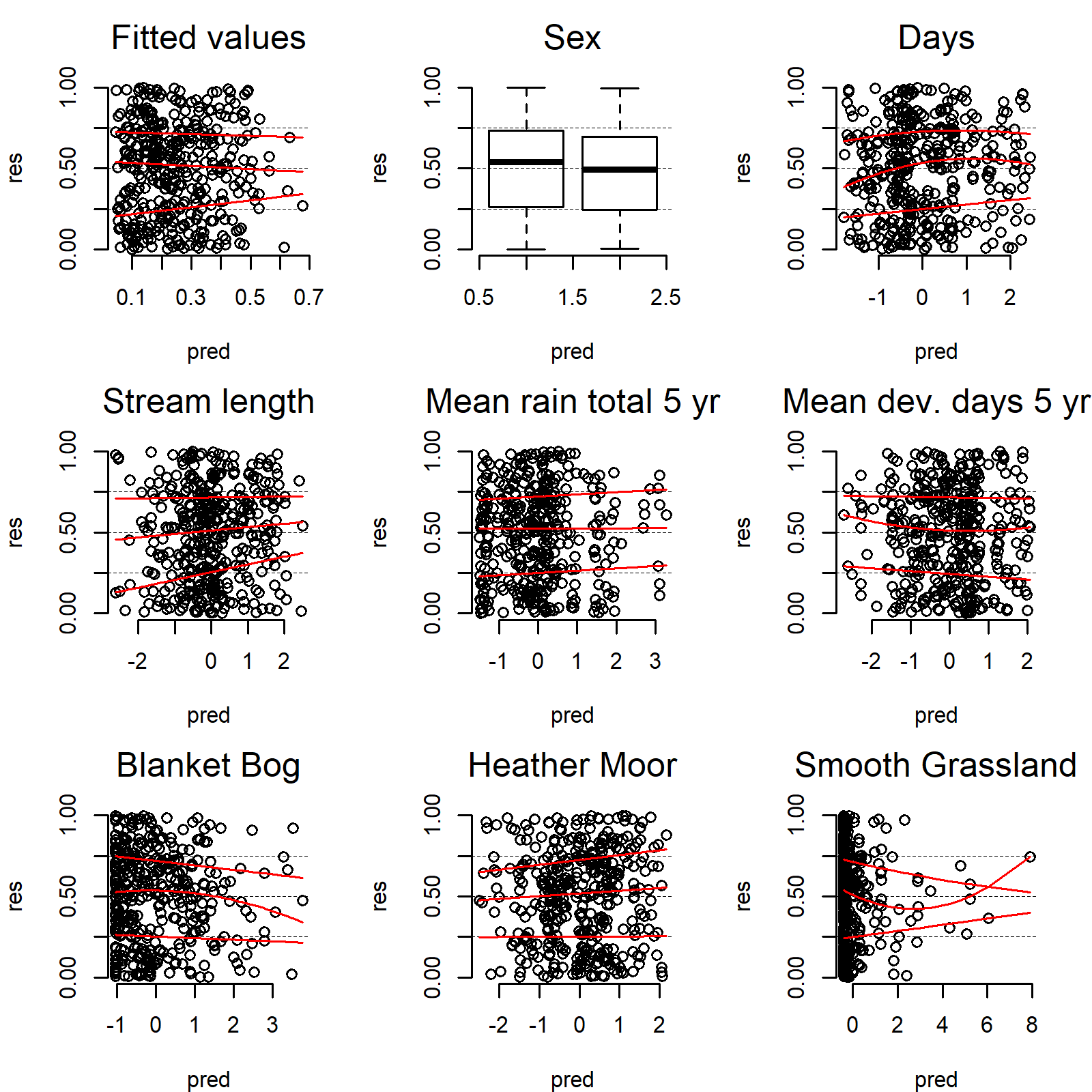

Supplement: Supplementary file 1 — Additional file 1: Figure S1. Standardized residuals vs predictors. This figure revealed no strong evidence of non-linear relationships between predictors and response (probability of diagnosis). The plot was produced using the plotResiduals() function in the DHARMa R package [43]. [file 13071_2019_3782_MOESM1_ESM.png]

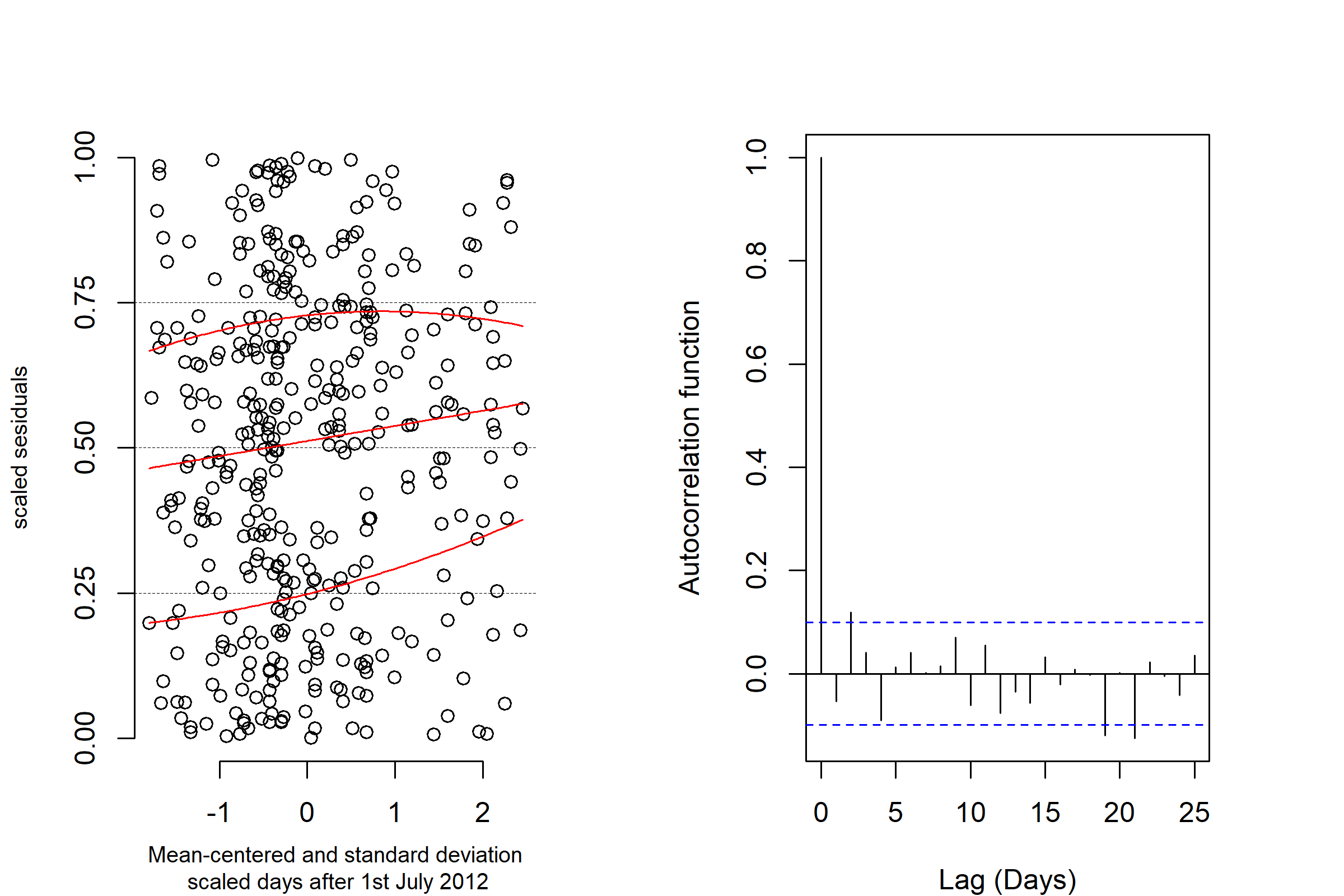

Supplement: Supplementary file 2 — Additional file 2: Residuals vs time (mean-centred and standard deviation scaled days after 1st July 2012) (left), autocorrelation function (ACF) for diagnostic data vs time lags of up to 25 days for model residuals (right). The dashed lines in the ACF plot illustrate the magnitude of the autocorrelation function beyond which autocorrelation is statistically significant. Therefore, this figure reveals borderline residual temporal autocorrelation at 1- and 8-days lag; neither of which we consider to be of concern. The figure was produced using the testTemporalAutocorrelation() function in the DHARMa R package [43] and the acf() function in the ncf R package [81]. [file 13071_2019_3782_MOESM2_ESM.png]

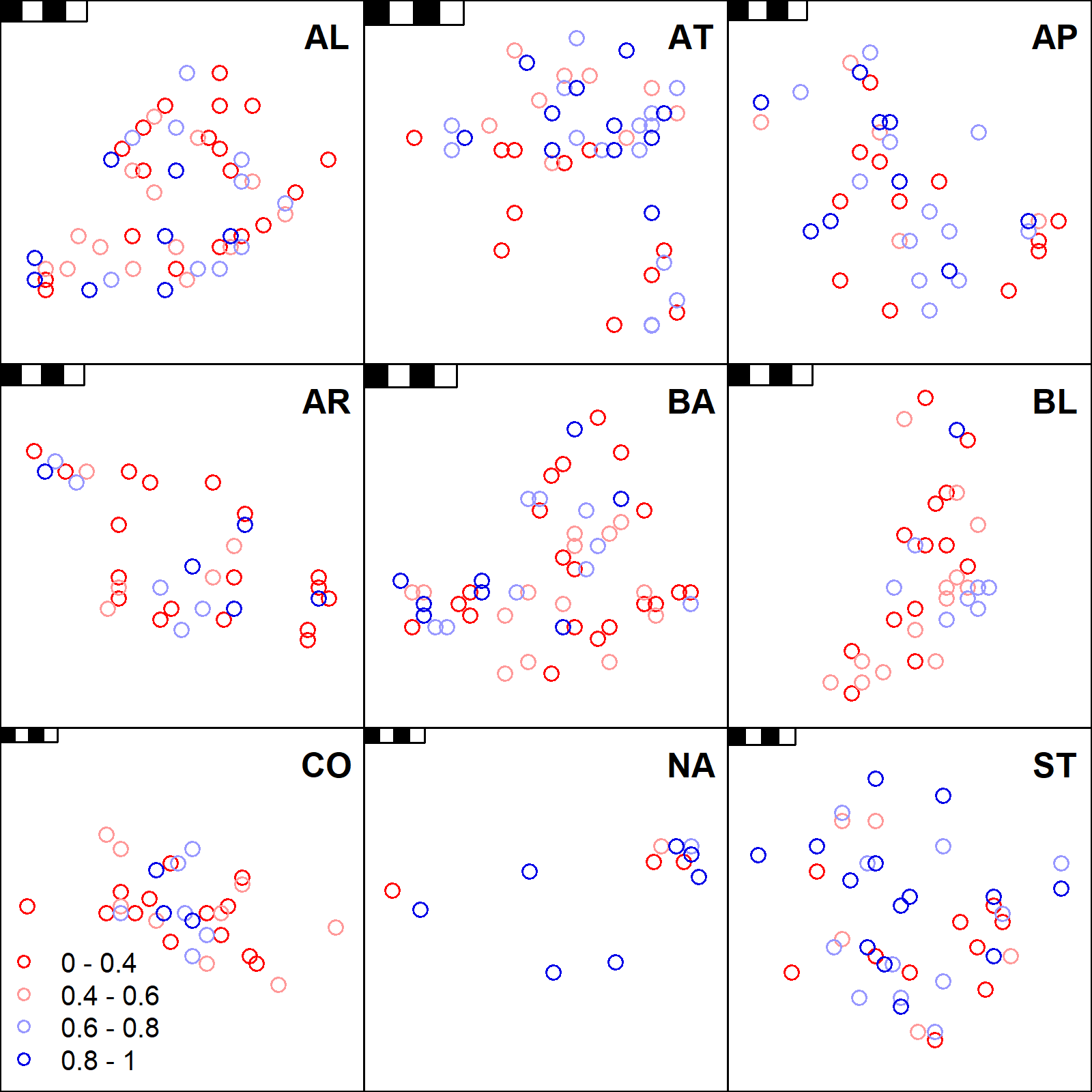

Supplement: Supplementary file 3 — Additional file 3: Figure S3. Spatially plotted residuals. This figure revealed no evidence of residual spatial autocorrelation and was created using an adaptation of the testSpatialAutocorrelation() function in the DHARMa R package [43]. The colour scale illustrates the magnitude of scaled simulated uniform residuals. [file 13071_2019_3782_MOESM3_ESM.png]
